# Identifying resting‐state effective connectivity abnormalities in drug‐naïve major depressive disorder diagnosis via graph convolutional networks

**DOI:** 10.1002/hbm.25175

**Published:** 2020-08-19

**Authors:** Eunji Jun, Kyoung‐Sae Na, Wooyoung Kang, Jiyeon Lee, Heung‐Il Suk, Byung‐Joo Ham

**Affiliations:** ^1^ Department of Brain and Cognitive Engineering Korea University Seoul Republic of Korea; ^2^ Department of Psychiatry Gachon University Gil Medical Center Incheon Republic of Korea; ^3^ Department of Biomedical Sciences Korea University College of Medicine Seoul Republic of Korea; ^4^ Department of Artificial Intelligence Korea University Seoul Republic of Korea; ^5^ Department of Psychiatry Korea University Anam Hospital, Korea University College of Medicine Seoul Republic of Korea

**Keywords:** effective connectivity, deep learning, graph convolutional networks (GCNs), major depressive disorder (MDD), resting‐state functional magnetic resonance imaging (rs‐fMRI), Sparse Group LASSO

## Abstract

Major depressive disorder (MDD) is a leading cause of disability; its symptoms interfere with social, occupational, interpersonal, and academic functioning. However, the diagnosis of MDD is still made by phenomenological approach. The advent of neuroimaging techniques allowed numerous studies to use resting‐state functional magnetic resonance imaging (rs‐fMRI) and estimate functional connectivity for brain‐disease identification. Recently, attempts have been made to investigate effective connectivity (EC) that represents causal relations among regions of interest. In the meantime, to identify meaningful phenotypes for clinical diagnosis, graph‐based approaches such as graph convolutional networks (GCNs) have been leveraged recently to explore complex pairwise similarities in imaging/nonimaging features among subjects. In this study, we validate the use of EC for MDD identification by estimating its measures via a group sparse representation along with a structured equation modeling approach in a whole‐brain data‐driven manner from rs‐fMRI. To distinguish drug‐naïve MDD patients from healthy controls, we utilize spectral GCNs based on a population graph to successfully integrate EC and nonimaging phenotypic information. Furthermore, we devise a novel sensitivity analysis method to investigate the discriminant connections for MDD identification in our trained GCNs. Our experimental results validated the effectiveness of our method in various scenarios, and we identified altered connectivities associated with the diagnosis of MDD.

## Graphical abstract

This study validated the use of effective connectivity (EC) for major depressive disorder (MDD) identification by estimating its measures via a group sparse representation along with a structured equation modeling approach in a whole‐brain data‐driven manner from resting‐state functional magnetic resonance imaging. To distinguish drug‐naive MDD patients from healthy controls, we utilize spectral graph convolutional networks (GCNs) based on a population graph to successfully integrate EC and nonimaging phenotypic information. Furthermore, we devise a novel sensitivity analysis method to investigate the discriminant connections for MDD identification in our trained GCNs.

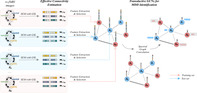

## 1 INTRODUCTION

Major depressive disorder (MDD), characterized by depressed mood, loss of interest, vegetative symptoms, and cognitive impairment, is a mental disorder that is prevalent worldwide (American Psychiatric Association, 2013). The lifetime prevalence of MDD was estimated to be 10.8% (American Psychiatric Association, 2013). The symptoms of MDD substantially interfere with social, occupational, interpersonal, and academic functioning (American Psychiatric Association, 2013). Globally, the total years lived with disability (YLD) of depressive disorders was 7.5% among all YLD, which has been ranked the highest of all disease (World Health Organization, 2017). Hence, depressive disorders are the leading cause of disability.

Despite the debilitating effects of MDD, the diagnosis of MDD is still made by phenomenological approach. Given the proximity to the psychiatric symptoms in terms of mood and cognitive dysregulation, brain MRI has been used to investigate the neural mechanisms of MDD (Kempton et al., 2011). Specifically, resting‐state functional magnetic resonance imaging (rs‐fMRI) has been widely used for the diagnosis of MDD by investigating altered functional networks while a subject is at rest (Anand et al., 2005; Craddock, Holtzheimer, Hu, & Mayberg, 2009; Greicius et al., 2007). In the meantime, more recently, the investigation of dynamic changes between connections beyond simple correlations has been attracting increasing interest (Geng, Xu, Liu, & Shi, 2018; Rolls et al., 2018). The notion of *effective connectivity* (EC) describes the influence of one neural system on another (Friston, Ungerleider, Jezzard, & Turner, 1994), in contrast to *functional connectivity* (FC) that denotes intrinsic correlations.

Several studies have revealed that the EC may be used as an efficient biomarker for the diagnosis of MDD. Specifically, (Schlösser et al., 2008) found that adolescents suffering from MDD exhibited a significant difference in EC between the amygdala and subgenual anterior cingulate cortex (ACC) during an emotion‐relevant task. In addition, Geng et al. (2018) directly utilized both FC and EC measures as features for the diagnosis of MDD and established that the discriminative power of EC features is higher than that of FC features. More recently, using a large sample size (336 patients with MDD and 350 control subjects), Rolls et al. (2018) identified significantly altered EC measures in MDD, such as reduced connectivity from temporal lobe areas to the medial orbitofrontal cortex. These findings imply that the EC measures are beneficial for determining if it is altered in neurological disorders, in addition to FC in the resting‐state paradigm in neuroimaging.

Several approaches such as dynamic causal modeling (DCM) (Park & Friston, 2013) and Granger causality (GC) (Granger, 1969) have been suggested for estimating EC. DCM is a commonly used approach; however, it requires the selection of seed regions of interest (ROIs) that are widely known as discriminant biomarkers in relevant literature rather than the whole brain connectivity due to computational complexity (Geng et al., 2018). GC, owing to its simplicity and ease of implementation, has been widely used to estimate the EC (Hamilton, Chen, Thomason, Schwartz, & Gotlib, 2011; Liao et al., 2011; Wu & Marinazzo, 2015). However, studies have shown that EC estimations given by GC cannot correctly determine the intensity of the actual causality in the time domain (Hu et al., 2012). In the meantime, structural equation modeling (SEM) (McIntosh, Rajah, & Lobaugh, 1999) has been successfully used as a statistical approach for investigating the EC (Büchel & Friston, 1997; Penny, Stephan, Mechelli, & Friston, 2004; Suk, Wee, Lee, & Shen, 2015; Wee, Yap, Zhang, Wang, & Shen, 2014; Zhuang, Peltier, He, LaConte, & Hu, 2008). The original work of SEM requires a large sample size to model complex relationships between brain activities.

In recent years, beyond the group‐level analyses, there has been growing interest in using machine learning (ML) techniques to identify clinically meaningful phenotypes for clinical diagnosis. A typical ML pipeline for the diagnosis of MDD can be summarized as follows: feature extraction, feature selection, model training, classification, and performance evaluation. In studies that differentiate MDD patients from healthy controls (HC), the following have been used as features extracted from rs‐fMRI: spatial independent components (Ramasubbu et al., 2016; Wei et al., 2013), the Hurst exponent (Jing et al., 2017), degree centrality (Li et al., 2017), and regional homogeneity (Ma, Li, Yu, He, & Li, 2013). In addition, many previous studies also applied graph theory approaches (Bhaumik et al., 2017; Cao et al., 2014; Drysdale et al., 2017; Guo et al., 2014; Lord, Horn, Breakspear, & Walter, 2012; Sundermann et al., 2017; Wang, Ren, & Zhang, 2017; Yoshida et al., 2017; Zeng, Shen, Liu, & Hu, 2014; Zhong et al., 2017) to the preestimated FC for investigating the disrupted functional brain networks in MDD patients. A small number of MDD classification studies have utilized EC as the feature. In Geng et al. (2018), EC was estimated using spectral DCM with predefined ROIs, and then, it was used as the feature for MDD classification; in this case, four supervised learning classifiers are used: linear support vector machine (SVM), nonlinear SVM, linear regression, and *k*‐nearest neighbor. Nonetheless, SVM (Bhaumik et al., 2017; Cao et al., 2014; Drysdale et al., 2017; Lord et al., 2012; Sundermann et al., 2017; Wang et al., 2017; Zhong et al., 2017) remains the most commonly used classifier, but other ML classifiers such as partial least squares regression (Yoshida et al., 2017), maximum margin clustering (Zeng et al., 2014), linear discriminant analysis (Ma et al., 2013), and neural networks (Guo et al., 2014) have also been applied for the diagnosis of MDD.

Recently, graph‐based approaches have gained popularity in medical applications owing to their ability to accommodate complex pairwise similarities in imaging/nonimaging features between subjects (Parisot et al., 2018). They model individuals as *vertices* and associations or similarities between them as *edges*, which have been widely used for supervised (e.g., classification (Tong et al., 2017)) and unsupervised tasks (e.g., manifold learning (Brosch & Tam, 2013; Wolz et al., 2012) and clustering (Parisot et al., 2016)). In this study, we focus on disease classification using a graph‐based model. In particular, a generalization of convolutional neural networks (CNNs) to an irregular graph domain, called spectral graph convolutional networks (GCNs), has been successfully applied to perform brain disease classification (Parisot et al., 2018). Specifically, (Parisot et al., 2018) utilized a population graph for GCNs, where a vertex represents a subject and an edge encodes pairwise similarities of phenotypic data and/or imaging features between subjects. This combines imaging and nonimaging data in a single framework and delivers competitive classification performance.

In this study, we go beyond the FC toward an EC‐based approach using a group sparse representation leveraged with SEM in an unsupervised manner. Specifically, this group‐constrained sparsity imposes similar connectional patterns among subjects but maintains individual differences in correlation weights. To identify MDD, inspired by Parisot et al. (2018), we exploit the spectral GCNs based on the population graph to successfully integrate our EC features and nonimaging demographic features. Furthermore, we devise a sensitivity analysis (SA) method for our learned GCNs to investigate discriminant EC measures for MDD identification. Through various scenarios, our experimental results validate the effectiveness of the proposed method in terms of extracted features, feature selection, and classifiers. Our main contributions can be summarized in two aspects as follows:

- We estimated EC by using a whole‐brain data‐driven approach with low computational costs through group‐constrained sparsity leveraged with SEM‐like mechanism and used it for the diagnosis of MDD via GCNs for the first time.
- In addition to superior experimental results for MDD identification, through an SA for our learned GCNs, we successfully identified meaningful connectivities associated with the diagnosis of MDD that have been reported in psychiatry literature.

## 2 MATERIALS

### 2.1 Participants

We collected the rs‐fMRI from 29 drug‐naïve MDD patients recruited from the outpatients of the Korea University Anam Hospital (Seoul, Republic of Korea). These patients included 8 males and 21 females; their ages ranged from 19 to 60 years, and the mean age was 43.79 years (±13.06). The outpatients were prospectively recruited as participants who agreed to visit the clinic after 4 weeks, 8 weeks, and 6 months. We defined drug‐naïve MDD patients based on the following two criteria: (a) those who were consistently diagnosed with MDD over the visits, and (b) those who had no record of prescribed medicine due to depressive symptoms at their first visit. The diagnosis was determined by board‐certified psychiatrists based on the Structured Clinical Interview from the Diagnostic and Statistical Manual of Mental Disorders, Fourth Edition (DSM‐IV) Axis I disorders. Basic demographic and clinical information such as family history of MDD and education level were acquired during the psychiatric interview at the clinic. The severities of depressive symptoms in all the participants were assessed using the 17‐item Hamilton Depression Rating Scale (HDRS‐17) (Hamilton, 1960) that reflects the degree of depression. The participants, at each visit, were assessed using the HDRS‐17, and MRI scanning was performed at the first visit.

A total of 44 HCs (17 males; 27 females) were recruited from the community; their ages ranged from 21 to 58 years. The recruitment was made with the help of an advertisement for those who voluntarily responded. The similar psychiatric diagnosis was carried out for HCs who were confirmed with none of any current symptoms and past history of psychiatric disorders. For both the groups, the participants who satisfied the criteria such as comorbidity of any other major psychiatric disorders, expressing psychotic features (i.e., delusion, hallucination), having a history of a serious or unstable medical illness including any primary neurological illness, and exhibited any contraindication to MRI scanning (e.g., metal implants) were considered inapplicable to the study. The protocol of the study was approved by the Institutional Review Board of Korea University Anam Hospital. In accordance with the Declaration of Helsinki, all the 73 participants signed a written informed consent prior to participating in the study. All participants were acknowledged thoroughly to drop out of the study at any stage, but there was no participant who dropped out. The demographic information is summarized in Table 1.

**TABLE 1:** Demographic information, psychiatric diagnosis and their statistical significance of MDD patients and HCs

	MDD (*n* = 29)	HC (*n* = 44)	*p*‐Value (*t*, *χ*^2^)
Age (years)	43.79 ± 13.06	39.68 ± 11.91	.169 (*t* = 1.389) ^a^
Gender (female/male)	21/8	27/17	.33 (*χ*^2^ = 0.948) ^b^
Education level			.018 (*χ*^2^ = 8.035) ^b^
Elementary and middle school	7	2	
High school or college/university	21	35	
Above graduate school	1	7	
HDRS‐17 score	14.48 ± 4.82	1.98 ± 2.11	<.001 (*t* = 13.166) ^a^

*Note:* Data presented as mean ± standard deviation or *n*, unless otherwise indicated.

Abbreviations: HC, healhy control; HDRS, Hamilton Depression Rating Scale; MDD, major depressive disorder.

^a^ Independent sample *t* test.

^b^ Pearson chi‐square.

There have been consistent evidences that patients with MDD had lower educational attainment as compared to HCs (Lorant et al., 2003). This means that lower educational level is one of the essential components of MDD which could not be separable from the diagnosis of MDD. So, in regard to the significant difference (*p*‐value = .018) between two groups in the education level, the distribution of the educational level between the two groups seems to appropriately reflect real‐world clinical situations. The unbalanced distribution of the educational level between the two groups would influence the classification results. However, there is no reason not to utilize nonneuroimaging data with neuroimaging data in one classification model. In clinical psychiatry, ML‐based approach primarily aims to build pragmatic model so that it can help psychiatrists to diagnose and treat mental disorders (Steele & Paulus, 2019). Hence, it is important to take full advantage of available data and maximize the performance of the classification model. In our method, we combine imaging and phenotypic data such as educational level in a single framework by constructing GCNs to enhance the classifying performance.

### 2.2 Data acquisition

Volumetric structural MRI scans were acquired using a 3.0 Tesla Siemens Trio whole‐body imaging system (Siemens Medical Systems, Iselin, NJ). A T1‐weighted magnetization‐prepared rapid gradient‐echo MP‐RAGE was used (repetition time [TR] = 1900 ms, echo time [TE] = 2.6 ms, field of view = 220 mm, matrix size = 256 × 256; 176 coronal slices without gap, voxel size = 0.9 × 0.9 × 1 mm^3^, flip angle = 9^∘^, and number of excitations = 1). Functional images were obtained using a single‐shot echo planer imaging sequence (TR = 2,000 ms, TE = 30 ms, flip angle = 90^∘^, number of slices = 42, matrix = 80 × 80, resolution = 3.0 × 3.0 × 3.0 mm^3^).

### 2.3 Preprocessing

We preprocessed data samples using the Data Processing Assistant for Resting‐State fMRI, a convenient plug‐in software based on SPM and REST. Among the 180 collected rs‐fMRI volumes, we initially discarded the first 10 volumes of each subject before any further processing to allow for magnetization equilibrium. Then, the remaining 170 volumes were slice‐timing corrected, head motion corrected, and spatially normalized to the standard Montreal Neurological Institute space with a resolution of 3 × 3 × 3 mm^3^. To further reduce the effects of nuisance signals, we performed the regressions of ventricle and white matter signals as well as six head‐motion profiles. Due to the controversy of removing the global signal in the postprocessing of rs‐fMRI data, we did not regress out the global signal. The regressed rs‐fMRI images were parcellated into 114 ROIs^1^ in the cortical regions, 57 per hemisphere, which are derived from the 17 networks using the functional atlas in Thomas Yeo et al. (2011). Subsequently, the mean rs‐fMRI time series at each ROI was computed and band‐pass filtered from 0.01 to 0.1 Hz to exploit the characteristics of low frequency fluctuations, thus resulting in a 114‐dimensional vector for each sample. Subjects with excessive head motion during scan acquisition^2^ were excluded from further analysis.

## 3 METHODS

**FIGURE 1:**
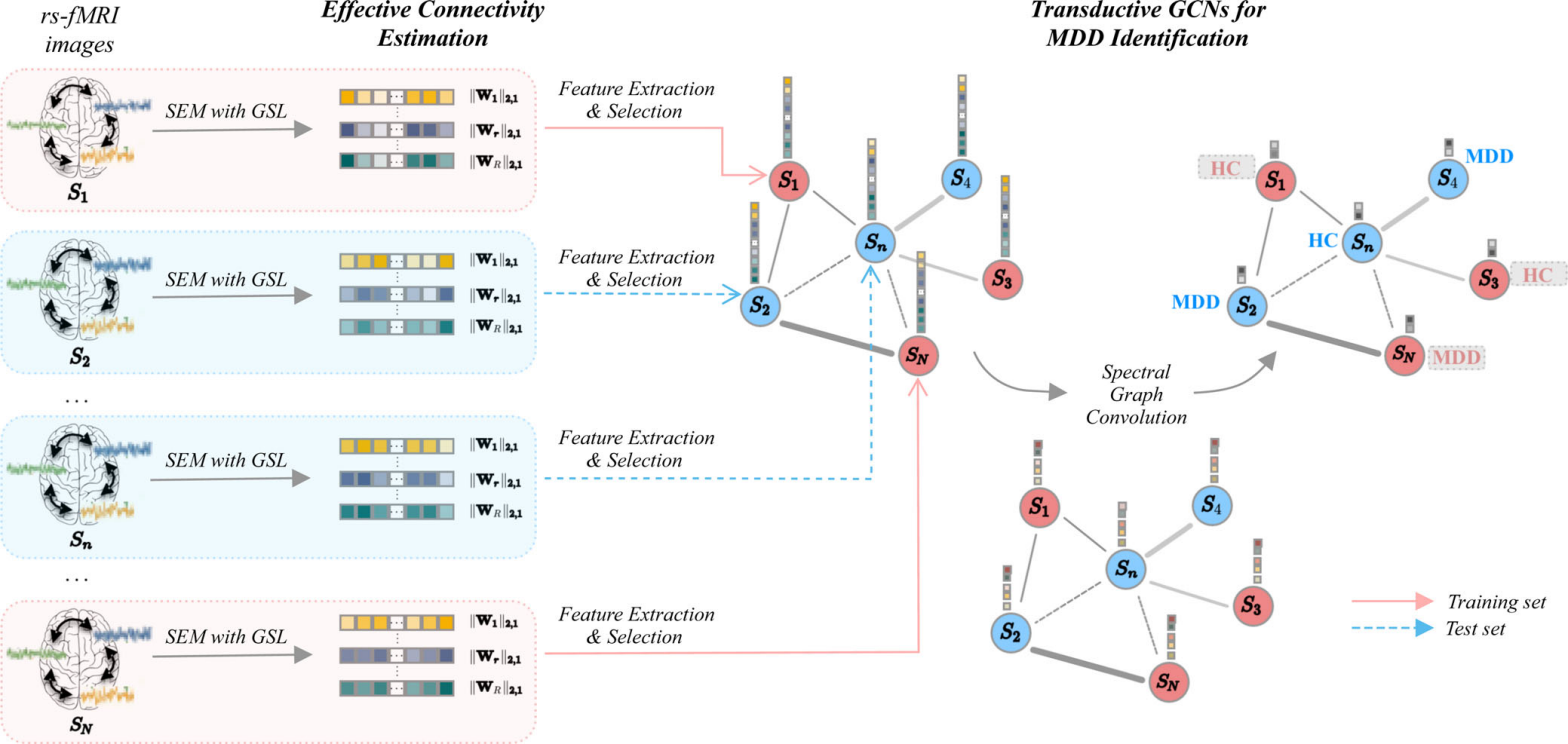
Overall framework of the proposed method for MDD identification. Test samples were marked with gray boxes to indicate that the test sample labels are never used during training. GCNs, graph convolutional networks; GSL, group‐constrained Sparse LASSO; MDD, major depressive disorder; SEM, structural equation model

In this section, we describe our experimental approaches for distinguishing drug‐naïve MDD patients from HCs based on rs‐fMRI time series. As shown in the overall procedure (Figure 1), we first estimate EC by a group sparse representation along with SEM in an unsupervised manner. This allows to impose similar connectional patterns among subjects but maintain individual differences in their network characteristics. We transform the estimated connectivity map into a vectorial feature space and further reduce its dimension based on statistically significant features while eliminating the redundant and less informative features in a univariate manner. The selected imaging feature vector and the phenotypic information (e.g., age, gender, etc.) of the subjects are incorporated into a population graph that forms the basis for our GCNs. A vertex represents each subject's acquisition, and an edge weight encodes the pairwise similarities of phenotypic information. By operating the spectral graph convolutions through the layers, the GCNs perform a binary classification between the MDD patients and HCs. In addition to MDD identification, we further introduce an SA method for our trained GCNs to detect discriminative EC measures.

### 3.1 Sparse estimation of EC

To estimate the fMRI‐derived features in the ML pipeline of MDD diagnosis, FC coefficients have been typically used (Bhaumik et al., 2017; Sundermann et al., 2017; Wang et al., 2017; Yoshida et al., 2017; Zhong et al., 2017). However, to validate the potential of the EC as a biomarker, we estimate the EC coefficients by leveraging the concept of SEM (Suk et al., 2015; Wee et al., 2014). Assume that a sequence of *T*‐length mean time series of rs‐fMRI from *R* ROIs is provided for subject *n*, that is, $X_{n}=\left[x_{n}^{1},\cdots ,x_{n}^{r},\cdots ,x_{n}^{R}\right]\in {\mathbb{R}}^{T\times R}$, where $x_{n}^{r}=\left[x_{n,1}^{r},\cdots ,x_{n,t}^{r},\cdots ,x_{n,T}^{r}\right]$ ^⊤^ ∈ℝ^*T*^. In this study, we hypothesize that the response of an ROI can be represented by a linear combination of those of other ROIs. That is, given the time course of the other ROIs excluding a target *r*th ROI, $X_{n}^{\backslash r}\in {\mathbb{R}}^{T\times \left(R-1\right)}$, we can formulate the time course of the target ROI as $x_{n}^{r}=X_{n}^{\backslash r}w_{n}^{\backslash r}+e$, where $w_{n}^{\backslash r}\in {\mathbb{R}}^{R-1}$ is a regression coefficient vector, and **e** is a zero‐mean Gaussian distributed error vector. It should be noted that these learnable regression coefficients of *N* subjects, $W_{1:N}^{\backslash r}=\left[w_{1}^{\backslash r},\cdots ,w_{n}^{\backslash r},\cdots ,w_{N}^{\backslash r}\right]\in {\mathbb{R}}^{\left(R-1\right)\times N}$, indicate the causal relations between a target ROI and the other ROIs.

Further, motivated by a recent study (Supekar, Menon, Rubin, Musen, & Greicius, 2008) that validated the effect of sparsity constraints for detecting robust connections from noisy connectivities, we apply a group‐constrained sparse least absolute shrinkage and selection (LASSO) (Wee, Yap, Zhang, Wang, & Shen, 2012) into our estimation of the EC. This sparse representation through ℓ_1_‐norm penalization can provide a biologically plausible interpretation, following the fact that a brain region typically forms relatively few numbers of connections. Hence, the objective function, ℒ(**W**^\*r*^), is defined as follows:

(1) $$ℒ\left(W_{1:N}^{\backslash r}\right)=\frac{1}{2}\sum_{n=1}^{N}\|x_{n}^{r}-X_{n}^{\backslash r}w_{n}^{\backslash r}{\|}_{2}^{2}+\alpha \|W_{1:N}^{\backslash r}{\|}_{2,1}$$

where *α* > 0 is a regularization parameter that indicates the magnitude of sparsity and ∥ ⋅ ∥_2,1_ denotes an ℓ_2,1_‐norm. The ℓ_2,1_‐norm is derived from the summation of ℓ_2_‐norms of $\|w_{n}^{\backslash r}{\|}_{1}$ that is an individually imposed ℓ_1_‐norm for each subject. This group‐constrained sparsity not only captures the consistent characteristics among subjects, but also retains intersubject variability. It is noteworthy that self‐to‐self connections are ignored by filling the *r*th element with zeros for each ROI, where we newly define ${\hat{W}}_{1:N}^{\backslash r}\in {\mathbb{R}}^{R\times N}$. The resulting unsupervised representation, ${\left\{{\hat{W}}_{1:N}^{\backslash r}\right\}}_{r=1}^{R}$, is regarded as the EC coefficients for all subjects.

Finally, we concatenate the estimated connectivities of all ROIs for a subject *n* such that $\left[{\hat{w}}_{n}^{\backslash 1},\cdots ,{\hat{w}}_{n}^{\backslash r},\cdots ,{\hat{w}}_{n}^{\backslash R}\right]\in {\mathbb{R}}^{R^{2}}$. Then, we conduct LASSO feature selection method to select informative features, thus resulting in **f**_*n*_ ∈ ℝ^*m*^, where *m* is a reduced dimension. Thus, a feature matrix for all *N* subjects, **F** = [**f**_1_, ⋯, **f**_*n*_, ⋯, **f**_*N*_] ^⊤^ ∈ℝ^*N* × *m*^, is fed into our classifier as the input.

### 3.2 Population graph construction

For classification, we use the GCNs (Parisot et al., 2018) based on a population graph. The population graph is represented as a weighted undirected graph $G=\left\{V,ℰ,W\right\}$, where V and ℰ are finite sets of ∣V∣=N vertices and edges respectively, and $W\in {\mathbb{R}}^{N\times N}$ denotes an weighted adjacency matrix. Specifically, each vertex corresponds to a subject and the edges encode the phenotypic similarities between every pair of subjects. To construct the aforementioned graph, the following two factors need to be determined: (a) the vertex feature vector assigned for each vertex and (b) the weighted adjacency matrix. In this study, we define **f**_*n*_ described in Section 3.1 as our feature vector for each vertex. Regarding the adjacency matrix, we consider the similarities of both imaging and nonimaging phenotypic features (e.g., age, gender) between subjects (Parisot et al., 2018). Given a set of *H* phenotypic measures $p_{n}={\left\{p_{n}^{h}\right\}}_{h=1}^{H}$ for subject *n*, each weight $W_{\mathrm{ij}}$ between subject *i* and *j* is defined as follows:

(2) $$W_{\mathrm{ij}}=\exp\left(-\frac{\|f_{i}-f_{j}{\|}^{2}}{2\sigma^{2}}\right)\sum_{h=1}^{H}\delta \left(p_{i}^{h},p_{j}^{h}\right)$$

where *σ* is a predefined kernel width of a Gaussian similarity function. With respect to *δ*(⋅), it depends on the type of phenotypic measure. For example, *δ*(⋅) is defined as the Kronecker delta function for categorical measures (e.g., subject's gender) or the unistep function for quantitative measures (e.g., subject's age) satisfying 1 iff $|p_{i}^{h}-p_{j}^{h}|<\gamma$; 0 otherwise, where *γ* is a threshold to be determined. Therefore, according to Equation (2), the edge weights increase when two subjects have a high similarity of vertex feature vectors and/or phenotypic measures. It is noteworthy that this population graph incorporates not only nonimaging features, but also imaging features, compared with many existing studies that use only imaging features for brain disease prediction.

### 3.3 Graph convolutional networks for MDD identification

After constructing the population graph represented in Section 3.2, we learn the GCNs to predict the target labels of MDD/HC. To this end, we introduce a spectral graph convolution as the main building block in GCNs, which generalizes the conventional convolution operation in the Euclidean domain to irregular graphs. It requires the eigen‐decomposition of the graph Laplacian (Chung & Graham, 1997) to be computed, followed by a graph Fourier transform (GFT) (Shuman, Narang, Frossard, Ortega, & Vandergheynst, 2013).

First, our population graph is represented by its Laplacian matrix ℒ, formulated as ℒ=D−W, where $D=\mathrm{diag}\left(d_{0},\ldots ,d_{N-1}\right)\in {\mathbb{R}}^{N\times N}$ is the diagonal degree matrix and $d_{i}=\sum_{j}W_{\mathrm{ij}}$ is the degree of vertex *i*. Because ℒ is a symmetric semidefinite matrix, it can be eigen‐decomposed such that ℒ = **U**Λ**U** ^⊤^, into a complete set of orthonormal eigenvectors **U** = [**u**_0_, …, **u**_*N* − 1_] ∈ ℝ^*N* × *N*^ and the diagonal matrix of nonnegative eigenvalues **Λ** = diag([*λ*_0_, …, *λ*_*N* − 1_]) ∈ ℝ^*N* × *N*^ (0 ≤ *λ*_0_ ≤ ⋯ ≤ *λ*_*N* − 1_). Particularly, it can be normalized as $ℒ=I_{N}-D^{-1/2}{\mathrm{WD}}^{-1/2}$, where **I**_*N*_ ∈ ℝ^*N* × *N*^ is an identity matrix, and the eigenvalues belong to the range of [−1, 1]. Accordingly, ℒ contains information about the connections between subjects and their similarities.

Following the property of the GFT, given vertex features **F** and a filter *g*_*θ*_ that is a diagonal matrix parameterized with Fourier coefficients *θ* ∈ ℝ^*N*^, the spectral convolutions are operated in the Fourier domain as *g*_*θ*_ * **F** = *g*_*θ*_(ℒ)**F** = *g*_*θ*_(**UΛU**^⊤^)**F** = **U***g*_*θ*_(Λ)**U** ^⊤^ **F**. Specifically, in this study, we apply filter approximation by representing *g*_*θ*_(**Λ**) as a *K*th order Chebyshev polynomial function of the eigenvalues (Defferrard, Bresson, & Vandergheynst, 2016; Hammond, Vandergheynst, & Gribonval, 2011), $g_{\theta }\left(\Lambda \right)=\sum_{k=0}^{K}\theta_{k}\Lambda^{k}$, where ${\left\{\theta_{k}\right\}}_{k=0}^{K}$ is a set of polynomial coefficients. This provides the benefits of *K*‐localization and cost‐effective computation of convolution. Thus, the convolution can be rewritten as follows:

(3) $$g_{\theta }*F=U\left(\sum_{k=0}^{K}\theta_{k}\Lambda^{k}\right)U^{⊤}F=\sum_{k=0}^{K}\theta_{k}\left(U\Lambda^{k}U^{⊤}\right)F=\sum_{k=0}^{K}\theta_{k}{ℒ}^{k}F.$$

On the basis of the spectral graph convolution, the overall model comprises multiple convolutional layers and a fully connected layer for the final prediction. In terms of the convolutional layer, layer‐wise activations are propagated, thus resulting in the representation of the *j*th output graph for the (*l* + 1)th layer activation from the *l*th layer activation, as follows:

(4) $${ℋ}_{j}^{\left(l+1\right)}=\sigma \left(\sum_{i=1}^{F_{\text{in}}}\left(\sum_{k=0}^{K}\theta_{{i,j}_{k}}{ℒ}^{k}{ℋ}_{i}^{\left(l\right)}\right)+b_{j}^{\left(l\right)}\right)$$

where *σ*(⋅) is a nonlinear activation function such as a rectified linear unit (ReLU) and $\theta_{{i,j}_{k}}$ is the (*F*_*in*_ × *F*_*out*_) vector of polynomial coefficients to be learned, and $b_{j}^{\left(l\right)}$ denotes the (1 × *F*_*out*_) bias vector in the *l*th layer. Here, we assume that by the GCN training, the vertices connected with high edge weights become more similar as they pass through multiple layers .

**FIGURE 2:**
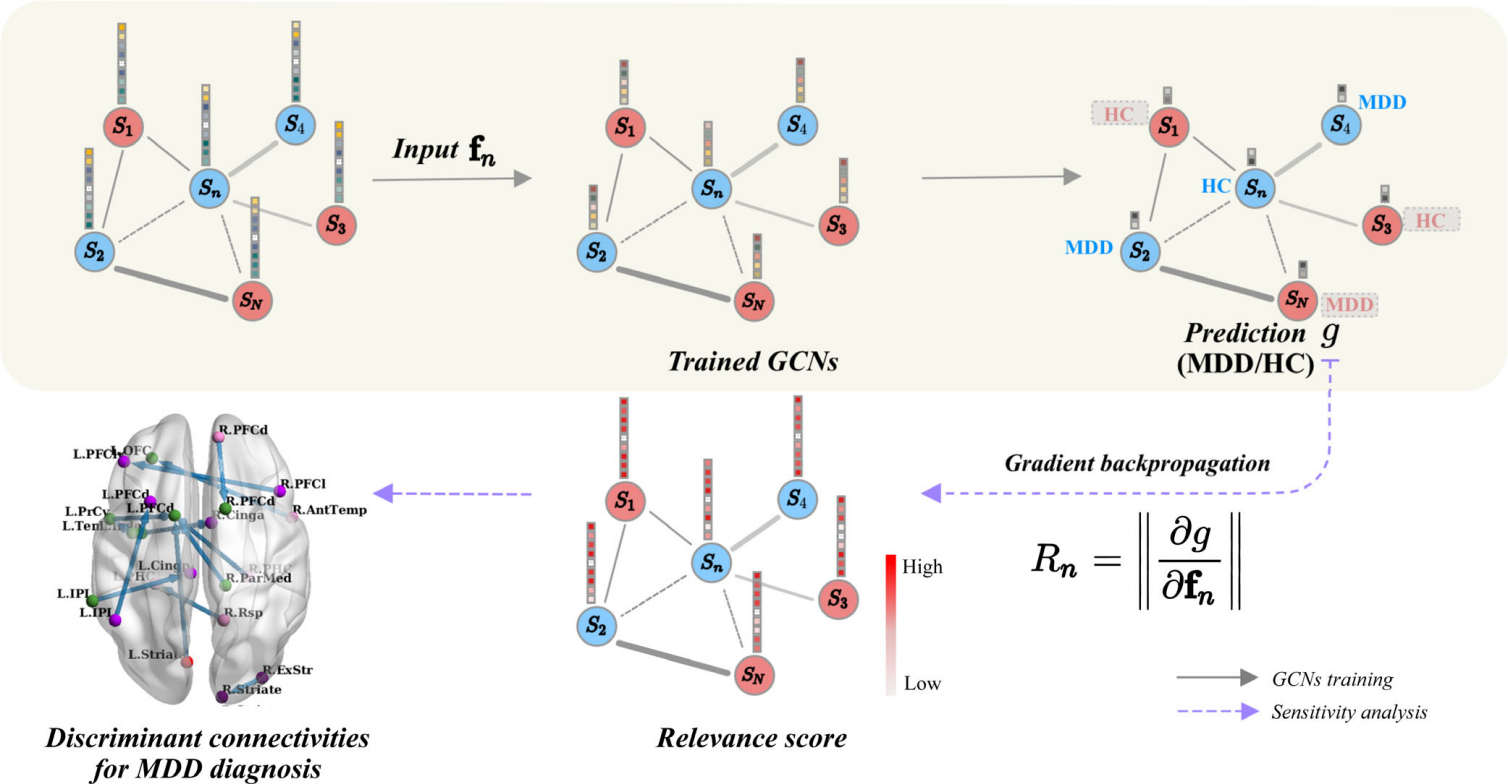
A schematic diagram of sensitivity analysis (SA) for our trained graph convolutional networks (GCNs). Gray lined arrows represent forward computation for major depressive disorder (MDD)/healthy control (HC) prediction, and purple dashed arrows denote gradient backpropagation of prediction with respect to input, resulting in the relevance scores

Finally, the final prediction layer comprises the fully connected layer followed by a softmax activation function. That is, the GCNs output a prediction label ${\hat{y}}_{n}$ that describes the brain state (e.g., MDD or HC) of a subject *n*. The loss function $J\left(\hat{y},y\right)$ is defined by the difference between $\hat{y}$ and the actual label **y** among test vertices, where a cross‐entropy loss function is used in our implementation. Basically, training the GCNs follows a transductive learning scheme. In other word, during the training, we use the whole data including labeled training and unlabeled test samples to construct the whole population graph. In addition, the features of test samples are exploited to perform the convolutions of training samples. The GCNs are trained to minimize the loss evaluated on the labeled training samples, and the parameters are updated by backpropagating the following two gradients:

(5) $$\frac{\partial J}{\partial \theta_{{i,j}_{k}}}={ℒ}^{k}{ℋ}_{i}^{\left(l\right)}\frac{\partial J}{\partial {ℋ}_{j}^{\left(l+1\right)}},\;\frac{\partial J}{\partial {ℋ}_{i}^{\left(l\right)}}=\sum_{j=1}^{F_{\text{out}}}\left(\frac{\partial J}{\partial {ℋ}_{j}^{\left(l+1\right)}}\left(\sum_{k=0}^{K}\theta_{{i,j}_{k}}{ℒ}^{k}\right)\right).$$

After training the GCNs, during the test, test samples are predicted with labels that maximize the probabilities of the softmax output.

### 3.4 Sensitivity analysis for interpretation of GCN‐based prediction

Many previous works have developed the methods to explain the predictions of deep learning models such as SA (Baehrens et al., 2010; Simonyan, Vedaldi, & Zisserman, 2013) and layer‐wise relevance‐propagation (Bach et al., 2015), and so forth. Recently, SA has been used in various applications such as medical diagnosis (Khan et al., 2001) and ecological modeling (Gevrey, Dimopoulos, & Lek, 2003), and so forth. However, to the best of our knowledge, interpretation techniques for GCNs have not been investigated yet. Thus, we devise a novel SA method for analyzing our trained GCN model. That is, in addition to the diagnosis, it provides an interpretation of what enables the GCNs to reach their individual predictions, thus allowing the identification of significantly altered EC measures in MDD patients.

SA is a gradient‐based model interpretation method. As shown in the Figure 2, it computes the norm ∥ ⋅ ∥_*q*_ over partial derivatives for a differentiable prediction function with respect to the input (i.e., a sensitivity of the prediction based on the changes in the input). Given our prediction function *g* and the vertex feature input **f**_*n*_ for subject *n*, relevance scores in SA are defined as follows:

(6) $$R_{n}={\left\|\frac{\partial g}{\partial f_{n}}\right\|}_{q}\in {\mathbb{R}}^{m}.$$

where ∥ ⋅ ∥_*q*_ is the norm of the partial derivative. To represent the magnitude to which variations of the input contribute to the output, the ℓ_1_ or ℓ_2_‐norm can be used (Kardynska & Smieja, 2016). A high relevance score implies that changes in the EC value influence the diagnosis of MDD significantly.

## 4 EXPERIMENTAL SETTINGS AND RESULTS

In this section, we validate the effectiveness of the proposed method for MDD identification by considering the following scenarios: (a) using FC or EC as features, (b) applying the feature selection or not, and (c) using GCNs or other ML method as a classifier. Furthermore, we identify the discriminant connectivities from the magnitude of resulting relevance scores in our SA method. All the codes are available at “https://github.com/ejju92/EC_GCN.”

### 4.1 Experimental settings

For performance evaluation, we took a 10‐fold stratified cross‐validation technique (Bishop, 2006). Specifically, we partitioned the samples of each class (i.e., drug‐naïve MDD patients and HCs) into 10 folds and used samples of 1 fold for testing and those of the remaining folds for training. Since we only have a total of 73 samples, including 29 drug‐naïve MDD patients and 44 HCs, that is, about 67 samples for the training set, we used the whole data including labeled training and unlabeled test set to construct population graph, as described in Section 3.3. However, the features of test set were used for the convolutions of training samples during training, and the loss is calculated only on a subset of training set. Note that the test sample labels were never used during training. As such, we repeated the above process 10 times by setting another different samples of 1 fold as the test set and rest as training set. The average of the results is reported in Section 4.2.

For constructing the population graph, we set *σ* = 1, *γ* = 2, and considered the ages and genders of the subjects as the phenotypic measures for adjacency matrix representation. We trained our GCNs with a single hidden layer that approximates the convolutions with third‐order Chebyshev polynomials, with parameters optimized by a grid search. For regularization, we applied dropout among the input, hidden, and prediction layers during training. The training hyper‐parameters are chosen as follows: a dropout rate of 0.3, a learning rate of 0.05, and an ℓ_2_ regularization of 5 × 10^−4^ with 200 epochs.

In this study, we considered comparable scenarios in terms of the feature type, feature selection, and classifier. For the extracted features, we compared FC and EC features. Many existing works (Azari et al., 1992; Van Dijk et al., 2009; Wang et al., 2007) have used the FC as a common measure of representative features from rs‐fMRI time‐series, demonstrating competitive performances in brain disease prediction tasks. Specifically, we estimated the FC by calculating pairwise Pearson correlation coefficients (Ye et al., 2015) between ROIs. Finally, we used its vectorized upper triangular part, thereby resulting in an *R*(*R* − 1)/2‐dimensional feature vector for each subject.^3^

In addition, we validated the effect of feature selection. Our feature vector is high dimensional with possibilities of including noisy features that may lead to performance degradation. Hence, we attempted to retain the features with the highest discrimination powers while eliminating redundant and less informative features using LASSO feature selection method.

To evaluate our proposed method, we compared it with other ML/deep learning methods. Regarding to the ML method, a linear SVM is exploited, which is a widely used classifier for brain disease diagnosis (Chen et al., 2016; Craddock et al., 2009; Fan et al., 2011). The SVM estimates an optimal hyperplane that best separates the two classes. We selected the model parameter *C* that balances between a regularization term in the set of {10^−5^, 10^−4^, …, 10^4^} by nested cross‐validation.

For the deep learning method, we evaluated BrainNetCNN (Kawahara et al., 2017) and discriminative/generative long short‐term memory (LSTM‐DG) (Dvornek, Li, Zhuang, & Duncan, 2019). The BrainNetCNN (Kawahara et al., 2017) is based on a CNN framework to capture the topological locality of structural brain networks. By taking the connectivity matrix as input, it uses novel edge‐to‐edge, edge‐to‐node, and node‐to‐graph convolutional filters for neurodevelopment prediction. With respect to the LSTM‐DG (Dvornek et al., 2019), i.e., joint LSTM‐DG network, it performs a multi‐task learning of brain disorder identification and rs‐fMRI time‐series data generation, given the rs‐fMRI ROI time‐series as input.

When calculating the relevance scores in the SA, we used the ℓ_1_‐norm that is the absolute of the partial derivative.

### 4.2 Performance results and analysis

For a quantitative evaluation of the comparable scenarios illustrated in Section 4.1, we considered the following metrics:

- ACCuracy (ACC) = (TP + TN)/(TP + TN + FP + FN).
- SENsitivity (SEN) = TP/(TP + FN).
- SPECificity (SPE) = TN/(TN + FP).
- Area under the curve (AUC).

where TP, TN, FP, and FN denote true positive, true negative, false positive, and false negative, respectively. Specifically, higher values of the sensitivity and specificity represent the lower chances of misdiagnosing each clinical label. We summarized the experimental results under various conditions in Table 2.

**TABLE 2:** Classification performance of various scenarios. The mean and *SD* over 10‐fold cross‐validation are represented. For each imaging feature, the highest performance is bolded in terms of each evaluation metric

Method	Metric	Effective connectivity	Functional connectivity
SVM	ACC	0.626 ± 0.144 ^a^	0.553 ± 0.252*
	SEN	0.266 ± 0.199 ^a^	0.350 ± 0.262*
	SPE	**0.870** ± **0.188** ^a^	0.690 ± 0.287*
	AUC	0.568 ± 0.156 ^a^	0.520 ± 0.249*
SVM w/LASSO	ACC	0.698 ± 0.104 ^a^	**0.603** ± **0.127** ^a^
	SEN	0.516 ± 0.216 ^a^	0.466 ± 0.266 ^a^
	SPE	0.825 ± 0.155 ^a^	0.710 ± 0.133 ^a^
	AUC	0.670 ± 0.110 ^a^	0.588 ± 0.146 ^a^
BrainNetCNN (Kawahara et al., 2017)	ACC	0.557 ± 0.103*	0.587 ± 0.153 ^a^
	SEN	0.200 ± 0.233*	0.433 ± 0.386 ^a^
	SPE	0.785 ± 0.248*	0.710 ± 0.245 ^a^
	AUC	0.492 ± 0.086*	0.571 ± 0.172 ^a^
LSTM‐DG (Dvornek et al., 2019)	ACC	0.564 ± 0.109*	
	SEN	0.333 ± 0.384*	
	SPE	0.745 ± 0.244*	
	AUC	0.539 ± 0.136*	
GCNs	ACC	0.591 ± 0.095*	0.539 ± 0.139*
	SEN	0.283 ± 0.258*	0.066 ± 0.133*
	SPE	0.820 ± 0.244*	0.850 ± 0.204*
	AUC	0.563 ± 0.211*	0.428 ± 0.168*
GCNs w/LASSO	ACC	**0.741** ± **0.130** ^b^	0.564 ± 0.140*
	SEN	**0.566** ± **0.300** ^b^	**0.466** ± 0.266*
	SPE	0.869 ± 0.166 ^b^	0.644 ± 0.217*
	AUC	**0.791** ± **0.153** ^b^	**0.665** ± 0.196*

*Note:* *: *p* < .05.

Abbreviations: ACC: ACCuracy; AUC, area under the curve; GCNs, graph convolutional networks; SEN, SENsitivity; SPE, SPECificity; SVM, support vector machine.

^a^ No statistical difference from the McNemar's test.

^b^ The reference method for the statistical tests with other methods.

As presented in Table 2, our method of GCNs w/LASSO demonstrated the best performance with respect to all the metrics, compared to other competitive methods including SVM, BrainNetCNN (Kawahara et al., 2017), and LSTM‐DG (Dvornek et al., 2019). From the experimental results, the following findings can be inferred: feature selection helps improve the performance in all scenarios. In particular, the effect of feature selection resulted in significant performance gains for high dimensional (*R*^2^) EC feature vector, which is approximately twice higher than that of FC (*R* × (*R* − 1)/2) given *R* ROIs. More specifically, the quantitative improvements for FC/EC in accuracy were 5.0/7.2% in SVM and 2.5/15% in GCNs, respectively.

In addition, the proposed method (GCNs w/LASSO) achieved the highest AUC in both EC and FC scenarios, implying that their predictions were not biased toward the majority class. It is noteworthy that in our dataset, because the number of samples available for each class was not balanced, that is, MDD patients (29) versus HC (44), the performance results could have been likely inflated. Nevertheless, our method achieved the AUC of 0.791 in EC and 0.665 in FC, respectively, demonstrating the power of our method to still identify the minority class well.

To demonstrate the statistical power of our method, we conducted a power (1‐probability of Type II error) analysis with R package (Kohl, 2019) that is based on a previous research (Flahault, Cadilhac, & Thomas, 2005). As shown in Table 2, the mean sensitivity (*SD*) of our classifier generated from 10‐fold cross‐validation is 0.566 ± 0.300. As the formula of a confidence interval is $\text{mean}\pm Z\frac{\mathrm{SD}}{\sqrt{n}}$, the mean sensitivity (95% CI) and marginal error is 0.566 (0.380–0.752) and 0.186, respectively. With *α* (probability of Type I error) = 0.05, sensitivity = 0.566, marginal error = 0.186, *Z* = 1.96, number of cases = 29, and number of controls = 44, the power of our classifier is estimated to 63.6%. When considering that most researchers set the statistical power to the range between 60 and 80% (OECD, 2014), the value of our statistical power is adequate.

In addition, in order to validate whether any observed difference between the proposed method and others is statistically significant, we conducted the McNemar' statistical test. We observed that the proposed method outperformed statistically (*p* − value < .05); the competing methods of BrainNetCNN (Kawahara et al., 2017) and GCNs for EC feature, SVM, GCNs, GCNs w/LASSO for FC feature, and LSTM‐DG (Dvornek et al., 2019).

We compared the computational time^4^ of the proposed method with that of our comparative methods in terms of training and test time (second) per epoch, as presented in Table 3. We measured the time on a NVIDIA GTX 1070 GPU. It is noteworthy that as our GCNs are tuning network parameters in a transductive manner, basically the learning process occurs in a testing phase only. Thus, the training and test time is identical.

**TABLE 3:** Comparison of the computational time between the proposed method and the competitive methods in terms of training and test time pear epoch

Measure	GCNs	SVM	BrainNetCNN (Kawahara et al., 2017)	LSTM‐DG (Dvornek et al., 2019)
Training time (s)	0.00375	0.00116	0.31375	0.21620
Test time (s)	0.00375	0.00015	2.18694	0.07804

Abbreviations: GCNs, graph convolutional networks; LSTM‐DG, discriminative/generative long short‐term memory; SVM, support vector machine.

Furthermore, we conducted a comparative experiment to estimate EC through GC analysis (GCA) for comparison with that of our proposed method. By using the estimated EC as feature, we performed MDD identification using GCNs, SVM, and BrainNetCNN (Kawahara et al., 2017) as classifier. The results are summarized in Table 4. It is noteworthy that with the GCA features, our proposed method was still superior to the competing methods in ACC, SEN, and AUC.

**TABLE 4:** Performance comparison between the case of using the GCA‐EC and ours. The mean and *SD* over 10‐fold cross‐validation are represented. For each method, the highest performance is bolded in terms of each evaluation measure

Method	Measure	GCA‐EC	Ours
SVM	ACC	0.576 ± 0.102	0.626 ± 0.144
	SEN	0.066 ± 0.133	0.266 ± 0.199
	SPE	**0.915** ± **0.187**	**0.870** ± **0.188**
	AUC	0.490 ± 0.077	0.568 ± 0.156
SVM w/LASSO	ACC	0.630 ± 0.081	0.698 ± 0.104
	SEN	0.233 ± 0.152	0.516 ± 0.216
	SPE	0.890 ± 0.142	0.825 ± 0.155
	AUC	0.561 ± 0.073	0.670 ± 0.110
BrainNetCNN (Kawahara et al., 2017)	ACC	0.519 ± 0.129	0.557 ± 0.103
	SEN	0.266 ± 0.409	0.200 ± 0.233
	SPE	0.720 ± 0.423	0.785 ± 0.248
	AUC	0.493 ± 0.078	0.492 ± 0.086
GCNs	ACC	0.498 ± 0.157	0.591 ± 0.095
	SEN	0.100 ± 0.152	0.283 ± 0.258
	SPE	0.760 ± 0.252	0.820 ± 0.244
	AUC	0.368 ± 0.244	0.563 ± 0.211
GCNs w/LASSO	ACC	**0.658** ± **0.187**	**0.741** ± **0.130**
	SEN	**0.633** ± **0.233**	**0.566** ± **0.300**
	SPE	0.684 ± 0.233	0.869 ± 0.166
	AUC	**0.738** ± **0.220**	**0.791** ± **0.153**

Abbreviations: ACC: ACCuracy; AUC, area under the curve; GCA‐EC, effective connectivity estimated by Granger causality analysis; GCNs, graph convolutional networks; SEN, SENsitivity; SPE, SPECificity; SVM, support vector machine.

### 4.3 SA‐based interpretation

**FIGURE 3:**
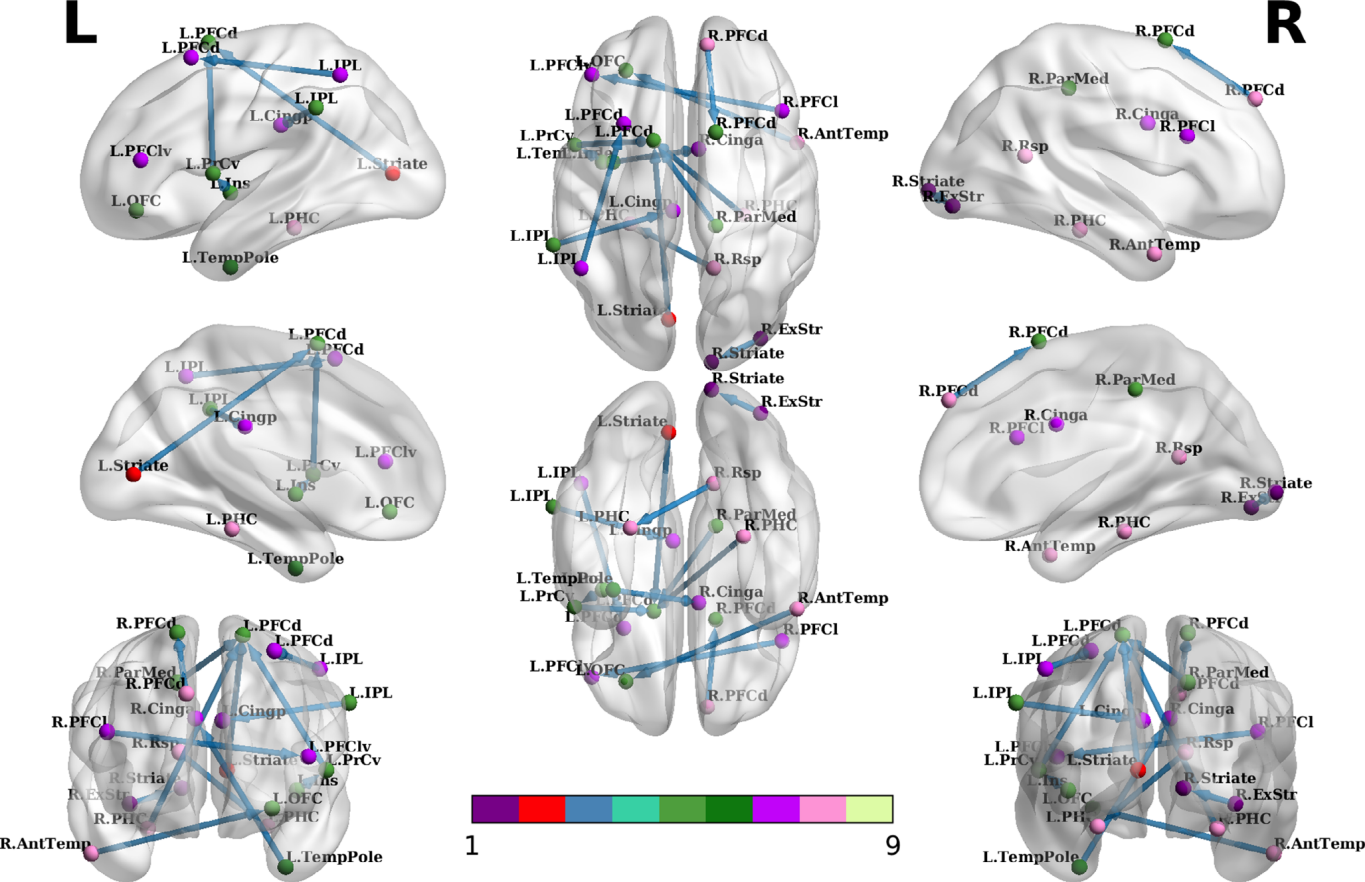
Discriminative effective connectivities from the sensitivity analysis (SA) of our graph convolutional networks (GCNs). Each color denotes the following brain networks: (1) central visual network, (2) peripheral visual network, (3) somatomotor network, (4) dorsal attention network, (5) salience/ventral attention network, (6) limbic network, (7) control network, (8) default network, and (9) temporal parietal network. All the above networks follow 17 brain networks defined in the study of Thomas Yeo et al. (2011)

As described in Section 3.4, we conducted the SA for our GCNs to identify significantly altered EC measures in MDD patients compared to HCs. From the SA, we obtained the relevance scores estimated for *N* subjects, $R={\left\{R_{n}\right\}}_{n=1}^{N}$. Here, after averaging them over all subjects, the mean relevance scores $\hat{R}$ were considered for analysis. Specifically, to investigate the discriminative EC measures, we selected the connectivities whose relevance scores were higher than (*μ* + 1.5 * *σ*), where *μ* and *σ* denote the mean and *SD* of the mean relevance scores, respectively. The selected connections are presented in Table 5 and Figure 3. The larger the relevance score values, the greater the importance of corresponding EC measures for the diagnosis of MDD.

**TABLE 5:** Discriminant effective connectivities from the SA of our GCNs. For each connection, we presented the index and name of the ROI, RS, and corresponding LASSO coefficient. The relevance scores are sorted in the descending order

Index	Source ROI	Index	Destination ROI	RS value	LASSO coefficient
19	Precentral ventral, left	24	Dorsal prefrontal cortex, left	0.99684	−1.19076
108	Anterior temporal, right	27	Orbital frontal cortex, left	0.97552	−0.01477
112	Retrosplenial, right	56	Parahippocampal cortex, left	0.92768	−0.75947
3	Striate cortex, left	24	Dorsal prefrontal cortex, left	0.82408	0.03766
79	Parietal medial, right	24	Dorsal prefrontal cortex, left	0.81150	−0.40647
38	Inferior parietal lobule, left	39	Dorsal prefrontal cortex, left	0.73504	0.68513
23	Inferior parietal lobule, left	44	Cingulate posterior, left	0.63800	−0.06180
59	Extrastriate cortex, right	58	Striate cortex, right	0.62140	0.72370
20	Insula, left	19	Precentral ventral, left	0.61601	0.31163
109	Dorsal prefrontal cortex, right	82	Dorsal prefrontal cortex, right	0.61421	0.37992
29	Temporal pole, left	94	Cingulate anterior, right	0.59003	−0.00683
113	Parahippocampal cortex, right	24	Dorsal prefrontal cortex, left	0.54320	0.19291
93	Lateral prefrontal cortex, right	35	Lateral ventral prefrontal cortex, left	0.51404	−0.41892

Abbreviations: GCNs, graph convolutional networks; ROI, region of interest; RS, relevance score; SA, sensitivity analysis.

Basically, we inputed the EC (EC) feature vector selected by our feature selection method, that is, LASSO, into the GCNs, and then applied SA to the learned GCN to investigate the discriminant connectivities for MDD identification from input feature vector. Through the LASSO feature selection, a total of 107 connectivities are selected from the 114 × 113/2 = 6,441 connectivities when considering the union of connectivities selected from all folds in cross‐validation, as shown in Table A2.

We examined the resulting LASSO coefficients for 13 connectivities chosen in the SA, as presented in Table 5. Considering that the mean coefficient for 107 connectivities is −0.00024, it is noteworthy that the coefficients for 13 connectivities have significantly high values and thus we believe that our GCNs well captured the informative features and their relations.

## 5 DISCUSSIONS

In this study, we successfully distinguished drug‐naïve MDD patients from HCs using GCNs. Hitherto, ML algorithms have been widely used for diagnosing MDD (Gao, Calhoun, & Sui, 2018). The accuracies of the performances ranged from good to excellent. For example, Lord et al. (Lord et al., 2012) and Wang et al. (Wang et al., 2017) reported 99.0 and 95.0% accuracy, respectively. Therefore, from the sheer number of reported accuracies, the difference in performance between ours and previous studies appears slight.

However, two distinguished features ensure the intrinsic reliability of our results. One is that we conducted a diagnostic evaluation of participants in the drug‐naïve state. Measuring neuroimaging materials in the drug‐naïve state is substantially important because drugs such as antidepressants have substantial effects on the structural (Dusi, Barlati, Vita, & Brambilla, 2015) and functional (Wessa & Lois, 2015) aspects of the brain. Another important methodological factor is that we ensured diagnostic stability for 6 months. Owing to the operational diagnostic criteria of the DSM series, diagnostic changes are not rare from a longitudinal perspective. For example, in the Korean population (Kim, Woo, Chae, & Bahk, 2011), the diagnostic consistency of MDD by DSM‐IV was only 84.8% in the first year. No matter how excellent the discriminating algorithms are, they are meaningless if the index diagnosis of MDD is changed to other indexes. To avoid the potential pitfall of cross‐sectional design, it is necessary to ensure longitudinal diagnostic stability. However, if the participation in the study is postponed until 1 or 2 years after the initial diagnosis, the confounding effects of the antidepressants can become problematic. Therefore, as suggested in a recent review (Kim & Na, 2018), we partially solved this issue using the MRI of participants whose diagnostic stability were confirmed for at least 6 months. Many previous ML studies did not provide reliable information of these critical methodological issues. Both the aforementioned studies that reported better discriminating performances than our results (Lord et al., 2012; Wang et al., 2017) did not mention the selection procedure of participants in terms of longitudinal diagnostic instability. Regarding antidepressants medication, one study reported that all the participants were taking antidepressants (Lord et al., 2012), and another study did not provide medication‐related information. We believe that the well‐defined selection process of the participants rendered our results more reliable than those of previously conducted studies.

### 5.1 Discriminative features analyses

Through the SA of our GCNs, we demonstrated that the dorsal prefrontal cortex received decreased connectivity from the precentral ventral, striate cortex, parietal medial, inferior parietal lobule, parahippocampal cortex. The dorsal prefrontal has long been known as a key region of depression, wherein cognitive reappraisal occurs in a top‐down manner (Alexander & Brown, 2011; Ochsner, Silvers, & Buhle, 2012). Disturbed connectivity with this region may result in biased selective attention to negative events and the related emotions such as depressive feeling, sadness, and shamefulness, which may contribute to the pathophysiology of MDD. However, the directions among the connectivities that contributed to the onset of depression have not been elucidated. By measuring the EC, we identified the directionality in the aberrant connectivity with this region.

Another interesting finding from the results of the SA is the abnormal connectivity from right retrosplenial cortices to the left parahippocampal cortices. The retrosplenial cortex is located in the posterior corpus callosum, the Brodmann areas 29 and 30. Meanwhile, the retrosplenial and parahippocampal cortices are jointly involved in visuospatial memory (Epstein, 2008; Mitchell, Czajkowski, Zhang, Jeffery, & Nelson, 2018); they are crucial in emotion regulation (Bubb, Kinnavane, & Aggleton, 2017; Maddock, 1999). Animal studies revealed that the retrosplenial cortex receives inputs primarily from the parahippocampal and prefrontal cortex (Sugar, Witter, van Strien, & Cappaert, 2011; Suzuki & Amaral, 1994). Indeed, the retrosplenial cortex is activated more than other regions in response to negative emotional words (Maddock & Buonocore, 1997). A possible mechanism by which the disturbed connectivity between the retrosplenial and parahippocampal cortices contribute to the MDD is through associative functions. Both the retrosplenial and parahippocampal cortices play a key role in the processing of contextual associations in MDD (Harel, Tennyson, Fava, & Bar, 2016). Broad scope and lively association exhibit a reciprocal relationship with positive mood and increased activity; narrow scope and ruminative pattern of thoughts tend to be associated with depressed mood, pessimistic thoughts of the future, and decreased energy (Bar, 2009; Harel et al., 2016; Nolen‐Hoeksema, 2000). We speculate that the decoupling of the retrosplenial and parahippocampal can result in inappropriate associative processing that, in turn, contributes to the negative view of future.

### 5.2 Limitations

This study has a few limitations that must be noted. First, the sample size (29 MDD patients and 44 HCs) may not be sufficiently large. Indeed, a recent study reported the characteristics of EC from the rs‐fMRI of MDD patients (*n* = 336) as compared to HC (*n* = 350) (Rolls et al., 2018). However, a fundamental difference exists between the previous study and our study. Whereas the previous study primarily examined the characteristics of EC in MDD via group‐level analysis, we aimed to discriminate MDD patients from HCs using the individual‐level approach. To the best of our knowledge, a GCN‐based deep learning model for distinguishing MDD patients from the HCs has not been developed. Second, detailed sociodemographic variables (e.g., marital status, cohabitation, and socioeconomic status) and clinical variables (e.g., current and past suicide attempt, family history of psychiatric disorder, and/or suicide death) were not fully obtained in the MDD group. Third, we discussed abnormal EC (e.g., disturbed bidirectional connectivity between parahippocampal and retrosplenial cortices) in relation with the characteristic symptoms of MDD (e.g., negative scope and rumination). However, we could not directly confirm such connections between EC and symptomatology in the case of MDD. Future studies require a larger sample size and relevant instruments for the investigation of symptoms.

## 6 CONCLUSION

In this study, we successfully estimated EC from rs‐fMRI and developed the GCN model for discriminating drug‐naïve MDD patients from HCs. We empirically exhibited the superiority of our method in various MDD classification scenarios, in terms of extracted features, feature selection, and classifiers. Because the performance ability did not provide any insight into the discriminant connectivity for the diagnosis of MDD, we devised a novel interpretation approach of our trained GCNs. Specifically, we applied the SA for the GCNs and selected the connectivities with high relevance scores. From the results of the SA, we could successfully identify regions that were previously identified as those associated with the MDD symptoms in the psychiatry literature. Thus, our results showed that EC may be promising for building deep learning‐based models in the field of neuroimaging. Further studies with a larger sample size are required to validate our findings.

## CONFLICT OF INTEREST

The authors declare no conflict of interest.

## ETHICS STATEMENT

This research obtained ethics approval from Korea University Anam Hospital, Seoul. All the participants agreed to join the research and gave informed consent before taking part.

## INFORMED CONSENT

In accordance with the Declaration of Helsinki, all the 73 participants signed a written informed consent prior to participating in the study.

## Data Availability

The data that support the findings of this study are available on request from the corresponding author (B. J. H.). The data are not publicly available due to restrictions, for example, their containing information that could compromise the privacy of research participants.

## Endnotes

^1^ For the names of all the regions, refer to Table A1.

^2^ We excluded patients with a displacement of greater than 2.5 mm and/or an angular rotation of greater than 2^∘^ in any direction.

^3^ In this paper, 114 × 113/2 = 6,441 dimensional vector.

^4^ The Python time module was used.

## Appendix 1

### Name of the ROIs in the Yeo template

**TABLE A1:** The index and name of the ROIs in the Yeo template (Thomas Yeo et al., 2011). The indices 1–57 and the indices 58–114 refer, respectively, to the left‐ and right‐hemispheric regions

Index	ROI label	Index	ROI label
1	Striate cortex (Striate)	58	Striate cortex (Striate)
2	Extrastriate cortex (ExStr)	59	Extrastriate cortex (ExStr)
3	Striate cortex (Striate)	60	Striate cortex (Striate)
4	Extrastriate inferior (ExStrInf)	61	Extrastriate inferior (ExStrInf)
5	Extrastriate superior (ExStrSup)	62	Extrastriate superior (ExStrSup)
6	Somatomotor A (SomMotA)	63	Somatomotor A (SomMotA)
7	Central (cent)	64	Central (cent)
8	S2 (S2)	65	S2 (S2)
9	Insula (Ins)	66	Insula (Ins)
10	Auditory (Aud)	67	Auditory (Aud)
11	Temporal occipital (TempOcc)	68	Temporal occipital (TempOcc)
12	Parietal occipital (ParOcc)	69	Parietal occipital (ParOcc)
13	Superior parietal lobule (SPL)	70	Superior parietal lobule (SPL)
14	Temporal occipital (TempOcc)	71	Temporal occipital (TempOcc)
15	Postcentral (PostC)	72	Postcentral (PostC)
16	Frontal eye fields (FEF)	73	Frontal eye fields (FEF)
17	Precentral ventral (PrCv)	74	Precentral ventral (PrCv)
18	Parietal operculum (ParOper)	75	Parietal operculum (ParOper)
19	Precentral ventral (PrCv)	76	Precentral (PrC)
20	Insula (Ins)	77	Precentral ventral (PrCv)
21	Parietal medial (ParMed)	78	Insula (Ins)
22	Frontal medial (FrMed)	79	Parietal medial (ParMed)
23	Inferior parietal lobule (IPL)	80	Frontal medial (FrMed)
24	Dorsal prefrontal cortex (PFCd)	81	Inferior parietal lobule (IPL)
25	Lateral prefrontal cortex (PFCl)	82	Dorsal prefrontal cortex (PFCd)
26	Ventral prefrontal cortex (PFCv)	83	Lateral prefrontal cortex (PFCl)
27	Orbital frontal cortex (OFC)	84	Lateral ventral prefrontal cortex (PFClv)
28	Medial posterior prefrontal cortex (PFCmp)	85	Ventral prefrontal cortex (PFCv)
29	Temporal pole (TempPole)	86	Medial posterior prefrontal cortex (PFCmp)
30	Orbital frontal cortex (OFC)	87	Cingulate anterior (Cinga)
31	Temporal (Temp)	88	Temporal pole (TempPole)
32	Intraparietal sulcus (IPS)	89	Orbital frontal cortex (OFC)
33	Dorsal prefrontal cortex (PFCd)	90	Temporal (Temp)
34	Lateral prefrontal cortex (PFCl)	91	Intraparietal sulcus (IPS)
35	Lateral ventral prefrontal cortex (PFClv)	92	Dorsal prefrontal cortex (PFCd)
36	Cingulate anterior (Cinga)	93	Lateral prefrontal cortex (PFCl)
37	Temporal (Temp)	94	Cingulate anterior (Cinga)
38	Inferior parietal lobule (IPL)	95	Temporal (Temp)
39	Dorsal prefrontal cortex (PFCd)	96	Inferior parietal lobule (IPL)
40	Lateral prefrontal cortex (PFCl)	97	Lateral dorsal prefrontal cortex (PFCld)
41	Lateral ventral prefrontal cortex (PFClv)	98	Lateral ventral prefrontal cortex (PFClv)
42	Medial posterior prefrontal cortex (PFCmp)	99	Medial posterior prefrontal cortex (PFCmp)
43	Precuneus (pCun)	100	Precuneus (pCun)
44	Cingulate posterior (Cingp)	101	Cingulate posterior (Cingp)
45	Inferior parietal lobule (IPL)	102	Temporal (Temp)
46	Dorsal prefrontal cortex (PFCd)	103	Inferior parietal lobule (IPL)
47	Posterior cingulate cortex (PCC)	104	Dorsal prefrontal cortex (PFCd)
48	Medial prefrontal cortex (PFCm)	105	Posterior cingulate cortex (PCC)
49	Temporal (Temp)	106	Medial prefrontal cortex (PFCm)
50	Inferior parietal lobule (IPL)	107	Temporal (Temp)
51	Dorsal prefrontal cortex (PFCd)	108	Anterior temporal (AntTemp)
52	Lateral prefrontal cortex (PFCl)	109	Dorsal prefrontal cortex (PFCd)
53	Ventral prefrontal cortex (PFCv)	110	Ventral prefrontal cortex (PFCv)
54	Inferior parietal lobule (IPL)	111	Inferior parietal lobule (IPL)
55	Retrosplenial (Rsp)	112	Retrosplenial (Rsp)
56	Parahippocampal cortex (PHC)	113	Parahippocampal cortex (PHC)
57	Temporal parietal (TempPar)	114	Temporal parietal (TempPar)

*Note:* Central visual network = (1–12, 58–59); peripheral visual network = (3–5, 60–63); somatomotor network = (6–10, 63–67); dorsal attention network = (11–17, 68–74); salience/ventral attention network = (18–28, 75–87); limbic = (29–30, 88–89); control network = (31–44, 90–101); default network = (45–56, 102–113); temporal parietal = (57, 114).

**TABLE A2:** Discriminant effective connectivities selected by LASSO feature selection method from all folds in cross‐validation. We highlighted the connectivities selected from sensitivity analysis. For corresponding connections, the index and name of the ROI are presented

Index	Source ROI	Index	Destination ROI
62	Extrastriate superior, right	5	Extrastriate superior, left
111	Inferior parietal lobule, right	54	Inferior parietal lobule, left
46	Dorsal prefrontal cortex, left	104	Dorsal prefrontal cortex, right
**38**	**Inferior parietal lobule, left**	**39**	**Dorsal prefrontal cortex, left**
41	Lateral ventral prefrontal cortex, left	39	Dorsal prefrontal cortex, left
**20**	**Insula, left**	**19**	**Precentral ventral, left**
35	Lateral ventral prefrontal cortex, left	84	Lateral ventral prefrontal cortex, right
54	Inferior parietal lobule, left	12	Parietal occipital, left
69	Parietal occipital, right	12	Parietal occipital, left
32	Intraparietal sulcus, left	91	Intraparietal sulcus, right
67	Auditory, right	66	Insula, right
79	Parietal medial, right	21	Parietal medial, left
88	Temporal pole, right	113	Parahippocampal cortex, right
103	Inferior parietal lobule, right	50	Inferior parietal lobule, left
48	Medial prefrontal cortex, left	46	Dorsal prefrontal cortex, left
39	Dorsal prefrontal cortex, left	82	Dorsal prefrontal cortex, right
**23**	**Inferior parietal lobule, left**	**44**	**Cingulate posterior, left**
82	Dorsal prefrontal cortex, right	39	Dorsal prefrontal cortex, left
97	Lateral dorsal prefrontal cortex, right	104	Dorsal prefrontal cortex, right
**3**	**Striate cortex, left**	**24**	**Dorsal prefrontal cortex, left**
6	Somatomotor A, left	15	Postcentral, left
75	Parietal operculum, right	84	Lateral ventral prefrontal cortex, right
34	Lateral prefrontal cortex, left	17	Precentral ventral, left
89	Orbital frontal cortex, right	30	Orbital frontal cortex, left
81	Inferior parietal lobule, right	75	Parietal operculum, right
15	Postcentral, left	6	Somatomotor A, left
16	Frontal eye fields, left	24	Dorsal prefrontal cortex, left
16	Frontal eye fields, left	33	Dorsal prefrontal cortex, left
**19**	**Precentral ventral, left**	**24**	**Dorsal prefrontal cortex, left**
77	Precentral ventral, right	19	Precentral ventral, left
22	Frontal medial, left	24	Dorsal prefrontal cortex, left
25	Lateral prefrontal cortex, left	24	Dorsal prefrontal cortex, left
3	Striate cortex, left	4	Extrastriate inferior, left
41	Lateral ventral prefrontal cortex, left	98	Lateral ventral prefrontal cortex, right
72	Postcentral, right	91	Intraparietal sulcus, right
20	Insula, left	78	Insula, right
80	Frontal medial, right	82	Dorsal prefrontal cortex, right
1	Striate cortex, left	58	Striate cortex, right
16	Frontal eye fields, left	22	Frontal medial, left
46	Dorsal prefrontal cortex, left	24	Dorsal prefrontal cortex, left
10	Auditory, left	67	Auditory, right
85	Ventral prefrontal cortex, right	26	Ventral prefrontal cortex, left
32	Intraparietal sulcus, left	13	Superior parietal lobule, left
112	Retrosplenial, right	55	Retrosplenial, left
18	Parietal operculum, left	94	Cingulate anterior, right
24	Dorsal prefrontal cortex, left	76	Precentral, right
47	Posterior cingulate cortex, left	105	Posterior cingulate cortex, right
**109**	**Dorsal prefrontal cortex, right**	**82**	**Dorsal prefrontal cortex, right**
**93**	**Lateral prefrontal cortex, right**	**35**	**Lateral ventral prefrontal cortex, left**
26	Ventral prefrontal cortex, left	85	Ventral prefrontal cortex, right
29	Temporal pole, left	76	Precentral, right
26	Ventral prefrontal cortex, left	94	Cingulate anterior, right
**29**	**Temporal pole, left**	**94**	**Cingulate anterior, right**
8	S2, left	65	S2, right
76	Precentral, right	24	Dorsal prefrontal cortex, left
68	Temporal occipital, right	69	Parietal occipital, right
**79**	**Parietal medial, right**	**24**	**Dorsal prefrontal cortex, left**
39	Dorsal prefrontal cortex, left	76	Precentral, right
82	Dorsal prefrontal cortex, right	24	Dorsal prefrontal cortex, left
98	Lateral ventral prefrontal cortex, right	89	Orbital frontal cortex, right
17	Precentral ventral, left	74	Precentral ventral, right
61	Extrastriate inferior, right	4	Extrastriate inferior, left
92	Dorsal prefrontal cortex, right	24	Dorsal prefrontal cortex, left
10	Auditory, left	9	Insula, left
92	Dorsal prefrontal cortex, right	33	Dorsal prefrontal cortex, left
**59**	**Extrastriate cortex, right**	**58**	**Striate cortex, right**
70	Superior parietal lobule, right	13	Superior parietal lobule, left
6	Somatomotor A, left	63	Somatomotor A, right
102	Temporal, right	24	Dorsal prefrontal cortex, left
99	Medial posterior prefrontal cortex, right	42	Medial posterior prefrontal cortex, left
55	Retrosplenial, left	112	Retrosplenial, right
17	Precentral ventral, left	27	Orbital frontal cortex, left
66	Insula, right	76	Precentral, right
91	Intraparietal sulcus, right	96	Inferior parietal lobule, right
24	Dorsal prefrontal cortex, left	27	Orbital frontal cortex, left
**113**	**Parahippocampal cortex, right**	**24**	**Dorsal prefrontal cortex, left**
77	Precentral ventral, right	76	Precentral, right
44	Cingulate posterior, left	101	Cingulate posterior, right
80	Frontal medial, right	76	Precentral, right
111	Inferior parietal lobule, right	69	Parietal occipital, right
80	Frontal medial, right	94	Cingulate anterior, right
4	Extrastriate inferior, left	61	Extrastriate inferior, right
41	Lateral ventral prefrontal cortex, left	27	Orbital frontal cortex, left
97	Lateral dorsal prefrontal cortex, right	40	Lateral prefrontal cortex, left
22	Frontal medial, left	16	Frontal eye fields, left
78	Insula, right	20	Insula, left
92	Dorsal prefrontal cortex, right	94	Cingulate anterior, right
13	Superior parietal lobule, left	70	Superior parietal lobule, right
98	Lateral ventral prefrontal cortex, right	76	Precentral, right
3	Striate cortex, left	5	Extrastriate superior, left
11	Temporal occipital, left	14	Temporal occipital, left
12	Parietal occipital, left	14	Temporal occipital, left
96	Inferior parietal lobule, right	38	Inferior parietal lobule, left
111	Inferior parietal lobule, right	103	Inferior parietal lobule, right
74	Precentral ventral, right	27	Orbital frontal cortex, left
11	Temporal occipital, left	68	Temporal occipital, right
82	Dorsal prefrontal cortex, right	27	Orbital frontal cortex, left
84	Lateral ventral prefrontal cortex, right	27	Orbital frontal cortex, left
23	Inferior parietal lobule, left	50	Inferior parietal lobule, left
75	Parietal operculum, right	81	Inferior parietal lobule, right
**112**	**Retrosplenial, right**	**56**	**Parahippocampal cortex, left**
60	Striate cortex, right	61	Extrastriate inferior, right
99	Medial posterior prefrontal cortex, right	27	Orbital frontal cortex, left
10	Auditory, left	57	Temporal parietal, left
14	Temporal occipital, left	57	Temporal parietal, left
45	Inferior parietal lobule, left	50	Inferior parietal lobule, left
**108**	**Anterior temporal, right**	**27**	**Orbital frontal cortex, left**
